# Haemostatic, fibrinolytic and inflammatory profiles in West Highland white terriers with canine idiopathic pulmonary fibrosis and controls

**DOI:** 10.1186/s12917-019-2134-z

**Published:** 2019-10-29

**Authors:** Elodie Roels, Natali Bauer, Christelle Lecut, Andreas Moritz, André Gothot, Cécile Clercx

**Affiliations:** 1 Department of Veterinary Clinical Sciences, FARAH, University of Liege, Avenue de Cureghem 3, 4000 Liège, Belgium; 2 Department of Veterinary Clinical Sciences, Clinical Pathophysiology and Clinical Pathology, Justus-Liebig University Giessen, Frankfurterstraße 126, 35392 Giessen, Germany; 3 Department of Haematobiology and Immunohematology, University Hospital of Liège, Avenue de l’Hôpital 13, 4000 Liège, Belgium

**Keywords:** Dogs, Lung, Clotting factors, Thromboelastometry, Coagulation

## Abstract

**Background:**

Canine idiopathic pulmonary fibrosis (CIPF) is a progressive interstitial lung disease mainly affecting old West Highland white terriers (WHWTs). The aetiology of CIPF is currently unknown and pathogenesis poorly understood. A genetic basis is strongly suspected based on the breed predisposition. CIPF shares clinical and pathological features with human IPF. In human IPF, coagulation disorders favouring a local and systemic pro-thrombotic state have been demonstrated in association with disease severity and outcome. The aim of this study was to compare the systemic haemostatic, fibrinolytic and inflammatory profiles of WHWTs affected with CIPF with breed-matched controls (CTRLs). Additionally, data collected in both groups were interpreted with regard to the reference intervals (when available) to assess possible pro-thrombotic features of the WHWT breed that may be related to CIPF predisposition. A total of 14 WHWTs affected with CIPF and 20 CTRLs were included.

**Results:**

WHWTs affected with CIPF had prolonged activated partial thromboplastine time in comparison with CTRLs (12.2 ± 0.9 s vs. 11.5 ± 0.7 s, *P* = 0.028), whereas results obtained in both groups were all within reference ranges. There was no significant difference between groups for the other factors assessed including plasmatic concentrations of fibrinogen, D-dimers concentration, antithrombin III activity, protein S and protein C activities, anti-factor Xa activity, activated protein C ratio, serum C-reactive protein concentration, and rotational thromboelastometry indices. Platelet count and plasmatic fibrinogen concentration were found to be above the upper limit of the reference range in almost half of the WHWTs included, independently of the disease status.

**Conclusions:**

Results of this study provide no clear evidence of an altered systemic haemostatic, fibrinolytic or inflammatory state in WHWTs affected with CIPF compared with CTRLs. The higher platelet counts and fibrinogen concentrations found in the WHWT breed may serve as predisposing factors for CIPF or simply reflect biological variation in this breed.

## Background

Canine idiopathic pulmonary fibrosis (CIPF) is a progressive fibrotic lung disease which is most commonly reported in aged dogs of the West Highland white terrier (WHWT) breed [1, 2]. CIPF share clinical, tomodensitometric and histopathological characteristics of both human IPF and other human interstitial lung diseases displaying non-specific interstitial pneumonia pattern [1–6]. In human idiopathic pulmonary fibrosis (IPF), an imbalance between thrombosis and fibrinolysis has been demonstrated in favour of a local and systemic pro-thrombotic state associated with disease severity and outcome [7, 8]. Extravascular coagulation involving fibrin formation in the intra-alveolar compartment has been proposed as a provisional matrix for migrating fibroblast contributing to pulmonary fibrosis [9]. Additionally, receptor-mediated actions of coagulant proteases on resident lung and infiltrating inflammatory cells have been suggested to play a role in fibrogenesis independently from fibrin formation mostly via protease-activated receptors (PARs) [9, 10]. Information about the role of the coagulation cascade in other human intestinal lung diseases has not yet been thoroughly studied. In CIPF, underlying pathophysiological mechanisms leading to fibrosis are still poorly elucidated despite growing research in recent years [2]. We hypothesized that CIPF in WHWTs is, at least partly, associated with a pro-thrombotic or pro-inflammatory state favouring lung fibrotic tissue deposition. The aim of this study was to assess blood biomarkers of haemostasis, fibrinolysis and inflammation in WHWTs affected with CIPF compared with breed-matched controls (CTRLs). Additional study objective was to compare data collected in both groups with reference intervals (when available) to assess possible pro-thrombotic features of the WHWT breed that might serve as predisposing factor for CIPF development.

## Results

### Animals

Details about the study population are summarized in Table 1. The CIPF diagnosis was achieved according to a previously published approach [1] and was confirmed by thoracic CT-scan alone (*n* = 6) or in combination with lung histopathology (*n* = 8). Control WHWTs were significantly younger than WHWTs affected with CIPF (*P* = 0.011). There was no difference between groups for sex repartition and body weight. Among CIPF WHWTs, 11/14 (79%) had a history of both exercise intolerance and coughing, and 3/14 (21%) exhibited only coughing at diagnosis. Crackles were noticed on lung auscultation in all dogs, a mild restrictive dyspnoea was present in 10/14 (71%) dogs and cyanosis was observed in 3/14 (21%) dogs. The duration of clinical signs at blood sampling ranged from 1.9 months to 4.2 years with a median of 1.4 years. Echocardiography was performed in all WHWTs at blood sampling. A tricuspid regurgitation jet was present in 9/14 (64%) dogs and indicated none (*n* = 1/9), mild (*n* = 5/9), or moderate (*n* = 3/9) pulmonary hypertension, with pulmonary systolic pressure gradient estimated at a median of 40.6 mmHg (range 15.9–64.0 mmHg, reference < 31.4 mmHg) [11]. Arterial blood gas analysis was performed in 7/14 (50%) dogs and revealed hypoxemia in all dogs with a median partial pressure of oxygen of 63 mmHg (range 58–77 mmHg, laboratory reference range: 80–100 mmHg). The 6-min walking test was performed in 10/14 (71%) CIPF WHWTs and a decreased walked distance was recorded in 5/10 dogs (median 378 m, range 198–524 m, reference > 420 m) [12]. At the time of blood sampling, 3/14 (21%) WHWTs affected with CIPF were treated with steroids (0.5–0.65 mg/kg q12-24 h PO), 3/14 (21%) with theophylline (10 mg/kg q8-12h PO), 3/14 (21%) with sildenafil (1 mg/kg q8-12h PO), 2/14 (14%) with N–acetylcysteine (15 mg/kg q12h PO), 1/14 (7%) with clopidogrel (2.5 mg/kg q24h PO), 1/14 (7%) with pimobendan (0.3 mg/kg q12h PO), and 1/14 (7%) with codeine (1 mg/kg q12h PO). Five (36%) CIPF WHWTs were untreated. Among control WHWTs included, 14/20 (70%) were clinically healthy; the remaining 6/20 (30%) dogs had presented for reasons unrelated to CIPF including post-operative recheck (1 month) following right ear conduct ablation, post-operative recheck (15 days) following rectal polyp resection, hip luxation, nasal tumor, bilateral otitis, and urinary incontinency respectively. Control dogs did not have any signs or findings indicating pulmonary disease. Echocardiography excluded the presence of primary cardiac disease in all control dogs. Thoracic high resolution computed tomography did not reveal significant abnormalities, except mild localized ground-glass opacity in the cranial lung lobes of 12/20 (60%) CTRLs. One control was treated with piroxicam (0.3 mg/kg q24h PO) and another with steroids (0.5 mg/kg q24h PO).

**Table 1:** Detailed characteristics of the study population

Variable	CIPF WHWTs	CTRL WHWTs	*P*-value
n	14	20	n.a.
Age, year	12.1 (7.4–15.4)	9.9 (5.7–15.0)	0.011
Body weight, kg	9.3 (6.2–17.7)	8.7 (6.6–11.0)	0.22
Sex, M/F	9/5	13/7	0.97

*Abbreviations*: *CIPF* canine idiopathic pulmonary fibrosis, *WHWTs* West Highland white terriers, *CTRL* control, *M* male, *F* female

### Haematological profile

Results of haematological profile obtained with the automated haematology analyser Cell Dyn 3700 from CIPF and CTRL WHWTs are summarized in Table 2. White blood cell count (WBC) and neutrophils count were significantly higher in WHWTs affected with CIPF in comparison with CTRLs (*P* = 0.0001), whereas WBC values above the upper limit of the reference range were found in only 3/12 (25%) CIPF and 2/19 (11%) CTRL dogs (*P* = 0.35). There was no difference in platelet count between groups, but PLT counts exceeded the upper limit of the reference range in 8/12 (67%) CIPF WHWTs and 7/19 (37%) CTRL WHWTs (*P* = 0.15).

**Table 2:** Haematological data from CIPF (*n* = 12) and CTRL (*n* = 19) WHWTs

Variable	CIPF WHWTs	CTRL WHWTs	*P*-value	RI
Hb (g/dL)	16.5 (11.3–19.3)	15.7 (12.6–18.2)	0.58	12–18
HCT (%)	48.7 (33.0–57.0)	46.8 (37.4–57.0)	0.96	37–55
RBC (10^12^ /L)	6.7 (4.6–8.3)	6.4 (4.9–8.0)	0.80	5.5–8.5
RDW (%)	16.7 (15.3–18.9)	16.5 (14.5–17.8)	0.21	14.3–19.7^a^
WBC (10^9^/L)	11.1 (9.0–23.6)	8.6 (3.4–21.6)	0.0001	6.0–15.0
Neutrophils (10^9^/L)	8.9 (5.7–19.2)	6.4 (1.6–19.2)	0.007	3.0–11.4
Monocytes (10^9^/L)	0.7 (0.07–2.2)	0.3 (0.01–1.4)	0.059	0.2–1.5
Lymphocytes (10^9^/L)	1.2 (0.7–2.6)	1.1 (0.0–3.5)	0.69	0.7–4.8
Eosinophils (10^9^/L)	0.1 (0.0–0.8)	0.09 (0.0–0.5)	0.67	0.1–1.5
PLT (10^9^/L)	539 (339–722)	411 (259–786)	0.12	200–500

*Abbreviations*: *CIPF* canine idiopathic pulmonary fibrosis, *WHWTs* West Highland white terriers, *CTRL* control, *RI* reference interval, *Hb* haemoglobin, *HCT* haematocrit, *RBC* red blood cell, *RDW* red cell distribution width, *WBC* white blood cell, *PLT* platelet

^a^Laboratory-internal reference intervals established from 111 clinically healthy dogs (unpublished data)

### Rotational thromboelastometry (ROTEM) profile

Results of ROTEM analysis are summarized in Table 3. There was no significant difference between groups for any of the parameters studied. Comparison with reference intervals was not possible as there were no laboratory-internal reference intervals validated in healthy dogs at the time of study writing.

**Table 3:** ROTEM data from CIPF (*n* = 10) and CTRL (*n* = 12) WHWTs

Variable	CIPF WHWTS	CTRL WHWTs	*P*-value
CT (sec)	48 (29–61)	42 (35–70)	0.24
∝-angle (°)	74 (69–77)	70 (57–76)	0.30
A30 (mm)	70 (62–82)	68 (53–78)	0.60
MCF (mm)	73 (63–82)	69 (55–78)	0.43
LY60 (%)	99 (93–100)	99 (96–100)	0.85
ML (%)	8 (0–14)	9 (3–43)	0.27

*Abbreviations*: *CIPF* canine idiopathic pulmonary fibrosis, *WHWTs* West Highland white terriers, *CTRL* control, *RI* reference interval, *CT* clotting time, *A30* amplitude at 30 min, *MCF* maximal clot firmness, *LY60* lysis after 60 min, *ML* maximal lysis. No laboratory-internal reference intervals available at the time of study writing

### STA compact profile

Results of the haemostatic profile analysis with the STA compact automated coagulation analyser are summarized in Table 4. WHWTs affected with CIPF demonstrated a slightly but significantly prolonged activated partial thromboplastine time (APTT) in comparison with CTRLs, whereas results were within reference interval for all dogs. Despite absence of significant difference between groups for the other variables, results outside the reference ranges for each individual parameter that may favour a hyper-coagulatory state were recorded and compared between groups. Results below the reference range were observed for protein S activity in 3/11 (27%) CIPF and 4/17 (24%) CTRLs (*P* = 1.000), for protein C activity in 1/11 (9%) CIPF and 2/17 (12%) CTRLs (P = 1.000), and for antithrombin III (AT-III) activity in 5/10 (50%) CIPF and 2/18 (11%) CTRLs (*P* = 0.063). Results above the reference range were observed for fibrinogen concentrations in 8/13 (62%) CIPF and 7/19 (37%) CTRLs (*P* = 0.28).

**Table 4:** Haemostatic data from CIPF and CTRL WHWTs

Variable	n	CIPF WHWTS	n	CTRL WHWTs	*P*-value	RI^a^
PT (sec)	13	7.2 (6.4–8.7)	19	7.2 (6.3–12.7)	0.48	5.7–8.1
APTT (sec)	12	12.1 (10.8–13.8)	16	11.4 (10.3–12.7)	0.028	10.0–14.3
Fibrinogen (g/L)	13	3.7 (1.9–8.5)	19	2.8 (1.6–5.1)	0.067	1.3–3.1
D-dimers (uGu/mL)	10	0.25 (0.07–0.32)	18	0.19 (0.09–0.30)	0.38	0.02–0.65
AT-III (%)	10	110 (82–215)	18	142 (96–171)	0.093	108–128
Protein S (%)	11	118 (23–274)	17	120 (0–496)	0.91	74.4–160.5
Protein C (%)	11	103 (72–286)	17	93 (10–304)	0.38	75.5–118.9
FXa (UI/mL)	10	0.10 (0.04–0.70)	18	0.16 (0.09–0.20)	0.29	0.04–0.26
APCR (sec)	11	25.6 (21.9–27.7)	17	26.8 (23.8–64.4)	0.066	20.0–30.0

*Abbreviations*: *CIPF* canine idiopathic pulmonary fibrosis, *WHWTs* West Highland white terriers, *CTRL* control, *RI* reference intervals, *PT* prothrombin time, *APTT* activated partial thromboplastine time, *AT-III* antithrombin III activity, *FXa* anti-factor Xa activity, *APCR* activated protein C ratio

^a^Laboratory-internal reference intervals established from 56 clinically healthy dogs (Bauer N [13])

For protein S, protein C and AT-III activities, the opposite scenario in favour of a hypo-coagulatory state was observed in some other WHWTs with results exceeding the reference range for protein S activity found in 4/11 (36%) CIPF and 4/17 (24%) CTRLs (*P* = 0.67), for protein C activity in 2/11 (18%) CIPF and 2/17 (12%) CTRLs (*P* = 1.000), and for AT-III activity in 3/10 (30%) CIPF and 15/18 (83%) CTRLs (*P* = 0.011). Anti-factor Xa activity was above the reference range in 3/10 (30%) CIPF and none of the controls (*P* = 0.037). Prothrombin time (PT) was prolonged in 1/13 (8%) CIPF and 1/19 (5%) CTRLs (P = 1.000).

### Serum C-reactive protein (CRP) concentration

There was no significant difference for serum CRP concentration between WHWTs affected with CIPF (median 3.7 nmol/L, range 1.1–56.1) and CTRL (3.1 nmol/L, 1.2–19.1). Results above 10 nmol/L indicative of an acute phase reaction were found in 1/13 CIPF (8%) and 2/16 (12.5%) CTRL (*P* = 1.000).

## Discussion

The present study investigated systemic parameters of haemostasis, fibrinolysis and inflammation in CIPF dogs compared with a breed-matched control group, hypothesizing the presence of a systemic pro-thrombotic or pro-inflammatory state in WHWTs affected with CIPF compared with CTRLs. Results obtained herein did not corroborate this hypothesis as no differences were observed between groups for the parameters studied, with the exception of APTT which was significantly prolonged in CIPF WHWTs. However, this was not considered as clinically relevant as it remained within the reference ranges in all dogs. An additional study objective was to compare individual data obtained in both groups with reference intervals, hypothesizing a possible pro-thrombotic state in the WHWT breed that may serve as predisposing factor for CIPF development and/or progression in a sub-category of dogs. Platelet count and plasmatic fibrinogen concentration were found to be above reference ranges in a substantial proportion of WHWTs in both groups which may favour this hypothesis. Results outside the reference ranges were also observed for protein S, protein C and AT-III activities, but were either below or above normal range precluding any reliable interpretation.

Human IPF is characterized by excessive interstitial deposition of extracellular matrix proteins by activated (myo) fibroblasts, resulting in reduced gas exchange and impaired pulmonary function [14, 15]. Animal model studies of fibrosis and human IPF patients have demonstrated a local imbalance between thrombosis and fibrinolysis within the alveolar compartment favoring fibrosis [7]. In addition to this local coagulation signalling dysregulation, a systemic pro-thrombotic state also occurs in IPF patients and has been associated with mortality and impaired lung function [16, 17]. In the study of Bargagli and collaborators (2014), pro-thrombotic state of stable IPF patients (*n* = 10) and those experiencing acute exacerbation (*n* = 23) was associated with increased serum concentrations of D-dimers, factor VIII activity, fibrinogen, and homocysteine compared with controls (*n* = 44), while there was no difference between groups for CRP concentrations, protein C and protein S activities, and clotting times [16]. Factor VII activity was also higher in cases of acute exacerbation in IPF patients who died following exabertion in comparison with patients who survived [16]. In a second study published by Navaratman and collaborators (2014), IPF patients (*n* = 211) were found to be four times more likely to have two or more clotting defects than controls (*n* = 256). Clotting defects taken into consideration included notably AT-III deficiency, increased factor VIII concentrations, protein C and free protein S deficiency, prolonged clot lysis time, and increases D-dimer concentration [17]. Pro-thrombotic state in IPF patients, defined as the presence of at least one clotting defect, was associated with disease severity at diagnosis measured by pulmonary function indices, and is associated with a three-fold increase in mortality [17].

The present study in dogs investigated systemic indicators of hemostasis, fibrinolysis and inflammation by the means of ROTEM analysis, clotting times measurement, and assessment of fibrinogen, D-dimers, AT-III, protein S, protein C, activated protein C ratio (APCR), anti-factor Xa activity (FXa) and CRP. Other systemic coagulation players such as factors VIII activity, or plasminogen concentration were not measured due to limited sample volume available for analysis. There was no relevant differences between CIPF and CTRL groups for the parameters studied, which may suggest a different underlying pathophysiology between canine and human pulmonary fibrosis. Interestingly, fibrinogen concentration was found to be above the reference ranges established from clinically healthy dogs of various breed (mostly large breed dogs) [13] in 8/13 (62%) CIPF WHWTs and 7/19 (37%) CTRLs (47% of the overall population). Fibrinogen is a positive acute phase protein which is converted by thrombin into fibrin to participate in blood clot formation [18]. Hyperfibrinogenaemia has been described in dogs in association with a hypercoagulable state in conditions such as neoplasia, infectious and inflammatory diseases [19–21]. Whether the high proportion of dogs with increased fibrinogen concentration found in this study represent normal biological breed variation or might be associated with a hypercoagulable state favouring the WHWT breed for CIPF or other disease condition is unknown. Similarly, haematological analysis revealed a platelet count above the upper limit of the reference range in 48% of the overall population of WHWTs included, which is in accordance with previous studies [22, 23]. In animal models of pulmonary fibrosis, platelets have been shown to accumulate within the lung and correlate with collagen deposition [24]. Recent studies in human IPF have shown increased platelet reactivity in affected patients, as measured by the aggregation of platelets with monocytes, platelet P-selectin expression, mean platelet volume and platelet binding to fibrinogen [25, 26]. Increased platelet reactivity in IPF may lead to pro-fibrotic mediator release contained within the alpha-granules such as platelet-derived growth factor or transforming growth factor-beta (TGF-beta) contributing to fibrosis [27]. TGF-beta signalling pathways have been studied in CIPF and associated with the pathogenesis of the disease [28, 29]. Whether the high platelet count observed in the WHWT breed may favour CIPF development or progression by facilitating TGF-beta fibrogenic action in a sub-category of dogs is unknown. White blood cell and neutrophil count were found significantly increased in CIPF dogs compared with CTRLs, while most results remained within the reference ranges. Some included CIPF WHWTs were on steroids administration at the time of blood sampling which may serve as an explanation for the increased white blood cell count. Leucocytosis may also be caused by a shifting movement of neutrophils from the marginating to the circulating pool secondary to stress from chronic disease, or by an increasing release from the bone marrow under the effect of inflammatory cytokines, such as chemokine (C-X-C motif) ligand 8 which has been shown to be increased in serum of WHWTs [30]. Among other haematological variables, the red blood cell distribution width (RDW) was recorded as this parameter has been shown to provide prognostic information in human IPF patients: RDW values above 15% at diagnosis are associated with a shorter survival time [31]. In dogs, increased RDW has been associated with severe pulmonary hypertension from pre- and post-capillary origin, but a considerable overlap in results with the control population has been noted [32, 33]. In the present study, there was no difference between CIPF and CTRL WHWTs for RDW, most likely because pulmonary hypertension was only mild to moderate in CIPF WHWTs included. Lastly, ROTEM analysis was performed to assess possible differences between groups for whole-blood coagulation. ROTEM takes into account both plasmatic and cellular elements of the coagulation and is the most useful tool to assess the presence of a hypercoagulable state [34]. There was no difference between CIPF and CTRL groups for neither of the ROTEM variables studied including parameters of clot formation kinetics, clot strength and fibrinolysis. Comparison with the human literature was not possible as there is presently no available study assessing ROTEM in human IPF nor other interstitial lung diseases. Comparison with reference intervals was not possible as validated laboratory-internal reference intervals established from a healthy population of dogs from various age and breed were not available at the time of study writing. ROTEM laboratory-internal reference intervals are recommended to be used instead of manufacturer intervals as multiple analytical and pre-analytical factors have been shown to influence ROTEM results in companion animals and in people [35].

The main limitation of this study was the small number of dogs included which may have impacted the statistical power of the analysis by increasing the risk of type II error. However, given the low prevalence of CIPF in the WHWT breed population, this patient series can be considered as relevant. Furthermore, the above mentioned study in human IPF displayed significant haemostatic changes compared with controls using a similar number of cases [16]. Another limitation was the absence of age matching between control and CIPF groups, and that some of the dogs were receiving treatment (e.g. corticosteroids) or had recent surgery at the time of blood sampling, which could have interfered with the results. Furthermore, 6/20 (30%) of the control dogs were not completely healthy as included in the study while presented for a localized problem independent of the cardiopulmonary system. However, when these dogs were assessed as a separate group, there was no change in the statistical analysis results (data not shown). Some control dogs also displayed mild localised ground-glass opacities in the cranial lung lobes which most likely reflect ventilation artefacts, whereas early subclinical CIPF lesions could not be completely rulled out. Lastly, the present study focused on systemic evidence of a pro-thrombotic and pro-inflammatory state in CIPF. Wheter blood data collected herein may or not reflect a local dysregulation of the coagulation and fibrinolytic pathways in situ within the lung tissue is unknown. Studies on bronchoalveolar lavage fluid and pulmonary tissue focusing on the coagulation and fibrinolysis cascades would be needed to gather additional data, but was out of the scope of the study.

## Conclusion

Results of the present study provided no clear evidence for a systemic pro-thrombotic or pro-inflammatory state in WHWTs affected with CIPF compared with breed-matched controls. Platelet count and plasmatic fibrinogen concentration were found above the upper limit of the reference range in a substantial proportion of WHWTs either CIPF or CTRL. Whether it may take part in the predisposition of the WHWT for CIPF or simply reflect biological variation in that breed remains to be elucidated.

## Methods

### Animals and study design

A total of 20 CTRLs and 14 CIPF WHWTs presented at the Small Animal Veterinary Clinic of the University of Liege in Belgium under the umbrella of the CIPF project (see: http://www.caninepulmonaryfibrosis.ulg.ac.be/ accessed 15.11.2018) between December 2013 and March 2016 were retrospectively selected for this study. In CTRLs, CIPF was ruled out by taking a complete history and by performing a physical examination, serum biochemistry, haematology, echocardiography and thoracic high resolution computed tomography which did not reveal significant abnormalities. Blood samples were collected by atraumatic venepuncture of the jugular vein using a 10 mL syringe and a 21-gauge needle. Cell blood count was prospectively measured in-house on whole-EDTA blood (Cell Dyn 3700, Abbott Diagnostics, GMI Inc.) in all included dogs immediately after the venepuncture, except for 3 dogs (2 CIPF and 1 CTRL) for which the blood was sent to a local external laboratory. Rotational thromboelastometry (ROTEM® Gamma, DSM Inc., Pentapharm GmbH) was prospectively run (ex-TEM profile) in duplicate on whole-citrated blood from 12 CTRLs and 10 CIPF WHWTs of the above mentioned population 30 min after the venepuncture at the Coagulation and Haemostasis Laboratory of the Human University Hospital of Liège, Belgium. ROTEM variables measured were clotting-time, *α*-angle, amplitude at 30 min, and maximal clot firmness. These parameters allow to assess to whole thrombotic capacity of the sample tested. Lysis after 60 min and maximal lysis were also recorded to investigate fibrinolytic properties of the sample. Remaining citrated blood was centrifuged twice at 10 °C for 10 min at 2500 x g, plasma was harvested and stored at − 80 °C until further analysis. Plain tubes were centrifuged after 30 min of collection at 4 °C for 15 min at 1300 x g, serum was harvested and stored at − 80 °C until further analysis. Frozen plasma and serum were shipped on dry ice to the department of Veterinary Clinical Sciences, Clinical Pathophysiology and Clinical Pathology of the Justus-Liebig University of Giessen, Germany within 3 years after sample collection for batch analysis. Coagulation times (PT and APTT), plasmatic concentrations of fibrinogen, D-dimer concentration, AT-III activity, protein S and protein C activities, FXa, and APCR were analysed in batch from plasma samples of all dogs using STA compact automated coagulation analyser as previously described [13]. These parameters were chosen to cover thrombotic (PT, APTT, fibrinogen, AT-III, protein S, protein C, FXa, APCR), fibrinolytic (D-dimers) and inflammatory (fibrinogen) plasmatic components of the blood. Plasma volume from 10 dogs (5 CIPF and 5 CTRLs) was insufficient to run all the parameters leading to partial results data. Serum CRP, an additional inflammatory marker, was measured in 16 CTRLs and 13 CIPF WHWTs of the above mentioned population on batch analysis using a validated dog-specific immunoturbidimetric CRP assay as previously described [36].

### Statistical analysis

Statistical analyses were performed using commercially available software (XLSTAT 2018 software, Addinsoft Inc). Continuous variables were reported as median and range (minimum and maximum), and categorical data as proportions and percentages. The Shapiro-Wilk test was applied to assess the distribution of continuous variables. Differences in continuous variables among CIPF and CTRL WHWTs were determined using student’s t-test (for normally distributed variables), or Mann-Whitney test (for variables that were not normally distributed). Proportions of dogs with results above or below the reference intervals were compared between groups using the Fisher exact test. For all analyses, *P-*value ≤0.05 was considered statistically significant.

## Data Availability

The datasets analysed during the current study are available from the corresponding author on request.

## Abbreviations

APCR: Activated protein C ratio
APTT: Activated partial thromboplastine time
AT-III: Antithrombin III
CIPF: Canine idiopathic pulmonary fibrosis
CRP: C-reactive protein
CTRLs: Controls
FXa: Anti-factor Xa activity
IPF: Idiopathic pulmonary fibrosis
PARs: Protease-activated receptors
PT: Pro-thrombin time
RDW: Red blood cell distribution width
ROTEM: Rotational thromboelastography
TGF-beta: Transforming growth factor beta
WBC: White blood cell count
WHWTs: West Highland white terriers

## References

[1] Heikkila-Laurila HP, Rajamaki MM (2014). Idiopathic pulmonary fibrosis in West Highland white terriers. Vet Clin North Am Small Anim Pract.

[2] Clercx C, Fastrès A, Roels E (2018). Idiopathic pulmonary fibrosis in West Highland white terriers: an update. Vet J.

[3] Roels E, Couvreur T, Farnir F, Clercx C, Verschakelen J, Bolen G (2017). Comparison between sedation and general anesthesia for high resolution computed tomographic characterization of canine idiopathic pulmonary fibrosis in West Highland white terriers. Vet Radiol Ultrasound.

[4] Thierry F, Handel I, Hammond G, King LG, Corcoran BM, Schwarz T (2017). Further characterization of computed tomographic and clinical features for staging and prognosis of idiopathic pulmonary fibrosis in West Highland white terriers. Vet Radiol Ultrasound.

[5] Johnson VS, Corcoran BM, Wotton PR, Schwarz T, Sullivan M (2005). Thoracic high-resolution computed tomographic findings in dogs with canine idiopathic pulmonary fibrosis. J Small Anim Pract.

[6] Syrja P, Heikkilä HP, Lilja-Maula L, Krafft E, Clercx C, Day MJ (2013). The histopathology of idiopathic pulmonary fibrosis in West Highland white terriers shares features of both non-specific interstitial pneumonia and usual interstitial pneumonia in man. J Comp Pathol.

[7] Crooks MG, Hart SP (2015). Coagulation and anticoagulation in idiopathic pulmonary fibrosis. Eur Respir Rev.

[8] Lin C, Borensztajn K, Spek CA (2017). Targeting coagulation factor receptors - protease-activated receptors in idiopathic pulmonary fibrosis. J Thromb Haemost.

[9] Schuliga M, Grainge C, Westall G, Knight D (2018). The fibrogenic actions of the coagulant and plasminogen activation systems in pulmonary fibrosis. Int J Biochem Cell Biol.

[10] Jose RJ, Williams AE, Chambers RC (2014). Proteinase-activated receptors in fibroproliferative lung disease. Thorax..

[11] Kellihan HB, Stepien RL (2010). Pulmonary hypertension in dogs: diagnosis and therapy. Vet Clin North Am Small Anim Pract.

[12] Lilja-Maula LI, Laurila HP, Syrjä P, Lappalainen AK, Krafft E, Clercx C (2014). Long-term outcome and use of 6-minute walk test in West Highland white terriers with idiopathic pulmonary fibrosis. J Vet Intern Med.

[13] Bauer N, Eralp O, Moritz A (2009). Reference intervals and method optimization for variables reflecting hypocoagulatory and hypercoagulatory states in dogs using the STA compact automated analyzer. J Vet Diagn Investig.

[14] Sgalla G, Iovene B, Calvello M, Ori M, Varone F, Richeldi L (2018). Idiopathic pulmonary fibrosis: pathogenesis and management. Respir Res.

[15] Barratt SL, Creamer A, Hayton C, Chaudhuri N (2018). Idiopathic pulmonary fibrosis (IPF): an overview. J Clin Med.

[16] Bargagli E, Madioni C, Bianchi N, Refini RM, Cappelli R, Rottoli P (2014). Serum analysis of coagulation factors in IPF and NSIP. Inflammation.

[17] Navaratnam V, Fogarty AW, McKeever T, Thompson N, Jenkins G, Johnson SR (2014). Presence of a prothrombotic state in people with idiopathic pulmonary fibrosis: a population-based case-control study. Thorax.

[18] Jeffery U, Staber J, LeVine D (2016). Using the laboratory to predict thrombosis in dogs: an achievable goal?. Vet J.

[19] Donahue SM, Brooks M, Otto CM (2011). Examination of hemostatic parameters to detect hypercoagulability in dogs with severe protein-losing nephropathy. J Vet Emerg Crit Care.

[20] McMichael MA, O’Brien M, Smith SA (2015). Hypercoagulability in dogs with blastomycosis. J Vet Intern Med.

[21] Vilar Saavedra P, Lara Garcia A, Zaldivar Lopez S, Couto G (2011). Hemostatic abnormalities in dogs with carcinoma: a thromboelastographic characterization of hypercoagulability. Vet J.

[22] Lawrence J, Chang YM, Szladovits B, Davison LJ, Garden OA (2013). Breed-specific hematological phenotypes in the dog: a natural resource for the genetic dissection of hematological parameters in a mammalian species. PLoS One.

[23] Heikkila HP, Lappalainen AK, Day MJ, Clercx C, Rajamäki MM (2011). Clinical, bronchoscopic, histopathologic, diagnostic imaging, and arterial oxygenation findings in West Highland white terriers with idiopathic pulmonary fibrosis. J Vet Intern Med.

[24] Piguet PF, Vesin C (1994). Pulmonary platelet trapping induced by bleomycin: correlation with fibrosis and involvement of the beta 2 integrins. Int J Exp Pathol.

[25] Ntolios P, Papanas N, Nena E, Boglou P, Koudelidis A, Tzouvelekis A (2016). Mean platelet volume as a surrogate marker for platelet activation in patients with idiopathic pulmonary fibrosis. Clin Appl Thromb Hemost.

[26] Crooks MG, Fahim A, Naseem KM, Morice AH, Hart SP (2014). Increased platelet reactivity in idiopathic pulmonary fibrosis is mediated by a plasma factor. PLoS One.

[27] Fernandez IE, Eickelberg O (2012). The impact of TGF-beta on lung fibrosis: from targeting to biomarkers. Proc Am Thorac Soc.

[28] Krafft E, Lybaert P, Roels E, Laurila HP, Rajamäki MM, Farnir F (2014). Transforming growth factor beta 1 activation, storage, and signaling pathways in idiopathic pulmonary fibrosis in dogs. J Vet Intern Med.

[29] Lilja-Maula L, Syrjä P, Laurila HP, Sutinen E, Rönty M, Koli K (2014). Comparative study of transforming growth factor-beta signalling and regulatory molecules in human and canine idiopathic pulmonary fibrosis. J Comp Pathol.

[30] Roels E, Krafft E, Antoine N, Farnir F, Laurila HP, Holopainen S (2015). Evaluation of chemokines CXCL8 and CCL2, serotonin, and vascular endothelial growth factor serum concentrations in healthy dogs from seven breeds with variable predisposition for canine idiopathic pulmonary fibrosis. Res Vet Sci.

[31] Nathan SD, Reffett T, Brown AW, Fischer CP, Shlobin OA, Ahmad S (2013). The red cell distribution width as a prognostic indicator in idiopathic pulmonary fibrosis. Chest.

[32] Swann JW, Sudunagunta S, Covey HL, English K, Hendricks A, Connolly DJ (2014). Evaluation of red cell distribution width in dogs with pulmonary hypertension. J Vet Cardiol.

[33] Mazzotta E, Guglielmini C, Menciotti G, Contiero B, Baron Toaldo M, Berlanda M (2016). Red blood cell distribution width, hematology, and serum biochemistry in dogs with echocardiographically estimated precapillary and postcapillary pulmonary arterial hypertension. J Vet Intern Med.

[34] McMichael MA, Smith SA (2011). Viscoelastic coagulation testing: technology, applications, and limitations. Vet Clin Pathol.

[35] Hanel RM, Chan DL, Conner B, Gauthier V, Holowaychuk M, Istvan S (2014). Systematic evaluation of evidence on veterinary viscoelastic testing part 4: definitions and data reporting. J Vet Emerg Crit Care.

[36] Gommeren K, Desmas I, Garcia A, Bauer N, Moritz A, Roth J (2018). Inflammatory cytokine and C-reactive protein concentrations in dogs with systemic inflammatory response syndrome. J Vet Emerg Crit Care.

